# Disaster-associated venous thromboembolism countermeasures in Japan: Insights from past major earthquakes

**DOI:** 10.1016/j.jvsv.2025.102312

**Published:** 2025-09-09

**Authors:** Keisuke Kamada, Eri Fukaya, Eriko Iwata, Daiki Uchida, Makoto Mo, Kazuhiko Hanzawa, Shinsaku Ueda, Nobuyoshi Azuma

**Affiliations:** a Department of Vascular Surgery, Asahikawa Medical University, Asahikawa, Hokkaido, Japan; b Disaster Countermeasure Committee, Japanese Society of Phlebology, Japan; c Division of Vascular Surgery, Stanford University School of Medicine, Stanford, CA; d Department of Cardiovascular Surgery, Nankai Medical Center Japan Community Healthcare Organization, Saeki, Oita, Japan; e Department of Vascular Surgery, Asahikawa City Hospital, Asahikawa, Hokkaido, Japan; f Department of Surgery, Yokohama City University, Yokohama, Kanagawa, Japan; g Department of Advanced Treatment and Prevention for Vascular Disease and Embolism, Niigata University Graduate School of Medical and Dental Sciences, Niigata, Niigata, Japan; h Healthcare Service Headquarters and Disaster Management and Social Welfare Department, Japanese Red Cross, Minato-ku, Tokyo, Japan

**Keywords:** Deep vein thrombosis, Venous thromboembolism, Venous screening, Earthquake, Disaster

## Abstract

Japan experiences more than 1000 perceptible earthquakes annually, including major events such as the 2011 Great East Japan Earthquake and the 1995 Great Hanshin-Awaji Earthquake. These events lead to a documented increase in cardiovascular events, particularly venous thromboembolism (VTE), including pulmonary embolism and deep vein thrombosis (DVT). Disaster-associated VTE is influenced by Virchow's triad: hemodynamic stasis, hypercoagulability, and endothelial injury. The incidence of DVT after earthquakes ranges from 10% to 30%, often developing 1 to 2 weeks post disaster. Early interventions in evacuation shelters to prevent venous stasis and hypercoagulability are critical. Left untreated, DVT can progress to pulmonary embolism, which may be fatal; however, most cases are preventable through timely intervention and improved shelter environments. Various organizations, including the Disaster Medical Assistance Team, the Japan Medical Association Team, and local institutions, contribute to disaster medical responses. In 2016, the Disaster Countermeasure Committee was established by the Japanese Society of Phlebology to lead VTE prevention efforts, including DVT screening, compression stocking distribution, public awareness campaigns, and promoting cardboard beds to enhance shelter conditions. In preparation for future large-scale disasters, it is vital to share evidence-based knowledge with health care professionals and the public to decrease the burden of disaster-associated VTE.

Japan is prone to earthquakes; the Japanese archipelago is located at the intersection of four tectonic plates. Approximately 1500 to 2000 perceptible earthquakes occur each year, including minor earthquakes. Oceanic plates are subducting beneath continental plates, generating significant strain that leads to frequent earthquakes, including devastating events such as the 2011 Great East Japan Earthquake, which was one of the most powerful earthquakes ever recorded (magnitude 9.0), triggering a massive tsunami. Additionally, numerous active faults exist within the interior of the country, causing intraplate earthquakes—such as the 1995 Great Hanshin-Awaji Earthquake and the 2016 Kumamoto Earthquakes—which often result in greater severe damage for their magnitude.

After major natural disasters such as floods, storms, typhoons, and earthquakes, there is an increase in the incidence of cardiovascular diseases, including hypertension,^1^ acute myocardial infarction,^2^ heart failure,^3^ pulmonary thromboembolism (PE),^4^ and deep vein thrombosis (DVT).^4^ Japan has experienced several large earthquakes in recent history, during which a notable number of cardiovascular events were reported. In particular, disaster-associated venous thromboembolism (VTE) gained widespread attention after the 2004 Mid-Niigata Prefecture Earthquake, owing to the high number of DVT cases and PE-related deaths.^5^ Currently, DVT and PE are also listed as reportable conditions in the Japan-Surveillance in Post Extreme Emergencies and Disasters system, the disaster medical reporting system used by emergency medical teams.

In evacuation shelters, many evacuees experience immobility owing to limited space, water supply shortages, reluctance to use public toilets, and physical trauma, factors that constitute risk conditions for DVT during earthquakes, based on Virchow's triad.^6^ DVT can lead to fatal PE; therefore, preventive intervention and early detection through venous screening are essential. In Japan, the Disaster Countermeasure Committee (DCC) was established by the Japanese Society of Phlebology in 2016 and has since been providing medical support and management during disasters. In this review, we describe our experience in disaster medicine and outline current challenges and future perspectives.

## Disaster and cardiovascular disease

### Cardiovascular diseases during disasters

The relationship between major disasters, such as earthquakes, and cardiovascular diseases has been well documented.^2^^,^^3^ The cardiovascular system is highly stress-sensitive organ systems, and its dysfunction may require immediate medical attention. Increases in acute coronary syndrome and Takotsubo cardiomyopathy were observed after the 1995 Great Hanshin-Awaji Earthquake and the 2004 Mid-Niigata Prefecture Earthquake.^7^^,^^8^ Similar findings have been reported in other countries; for instance, the Northridge earthquake in the United States was associated with numerous cases of sudden cardiac death.^9^

Stress is a critical contributor to a wide range of cardiovascular disorders, which can be categorized into acute and chronic phases. Acute stress activates the sympathetic nervous system and the coagulation cascade, and induces vascular reactivity abnormalities.^10^ Stressors may include physical, biological, chemical, and psychological factors, but these typically converge into persistent psychological stress during the chronic phase.

The disaster cardiovascular prevention risk score (AFHCHDC7) and prevention score (SEDWITMP8) have been proposed as tools for risk assessment and management during disasters.^11^ As shown in Table I, AFHCHDC7 includes seven factors, each set at 1 point; the total score of 4 or higher indicates a high-risk for individuals. SEDWITMP8 has eight factors, each set at 1 point; a score of 6 or higher is the target prevention score for individuals and shelters. These scoring systems are effective for preventing cardiovascular diseases during disasters, because they provide clear and individual goals for risk management and facilitate information sharing between shelters and medical institutions.

**Table I:** Disaster cardiovascular prevention (*DCAP*) risk score (*AFHCHDC7*) and prevention score (*SEDWITMP8*)

DCAP - AFHCHDC7 risk score		
1	Age (A)	>75 years
2	Family (F)	Death or hospitalization (partner, parents, or children)
3	Housing (H)	Completely destroyed
4	Community (C)	Completely destroyed
5	Hypertension (H)	Positive (under medication, or systolic blood pressure >160 mmHg)
6	Diabetes (D)	Positive
7	Cardiovascular disease (C)	Positive (coronary artery disease, stroke, heart failure)
DCAP - SEDWITMP8 Prevention Score		
1	Sleep (S)	Sleep duration >6 hours, arousal <3 times during sleep
2	Physical activity (E)	Walking >20 minutes/day
3	Diet (D)	Reduce salt intake with high potassium intake (3 serves of green vegetable, fruit, or seaweed/day)
4	Body weight (W)	Change < ±2 kg
5	Infection prevention (I)	Regular face mask use and washing hands
6	Thrombosis (T)	Sufficient water intake >1000 mL/day
7	Medication (M)	Continuous use of antihypertensive medication and antiplatelet agents and/or anticoagulation
8	Blood pressure control (P)	<140 mm Hg systolic (clinic, salter, or self-measured)

### Disaster-associated VTE

Disaster-associated VTE has been widely recognized in Japan since the 2004 Mid-Niigata Prefecture earthquake, during which numerous cases of DVT and PE-related deaths were reported.^4^ This finding was linked to the widespread practice of evacuees spending nights in their vehicles. After this event, DVT screening has been actively implemented in evacuation shelters, and knowledge regarding VTE in disaster settings has accumulated. Table II shows the incidence rates of DVT in major earthquakes over the past two decades.^5^^,^^12^, ^13^, ^14^, ^15^, ^16^, ^17^, ^18^ Although DVT incidence during earthquakes ranges from approximately 10% to 30%, this represents a significantly higher incidence than the estimated 2.5% incidence in the general population.^19^

**Table II:** The incidence rate of deep vein thrombosis (*DVT*) in major earthquakes in the past two decades

Earthquake (year)	Maximum magnitude	Sample size (n)	Incidence rate of DVT	Follow-up	Location	First author
Mid-Niigata prefecture (2004)	6.7	69	31.8%	2-3 weeks	Shelters	Hanzawa^5^
		82	11.7%	4-8 weeks	Shelters	
Noto peninsula (2007)	6.9	212	10.6%	N/A	Shelters	Terakami^12^
Iwate-Miyagi Nairiku (2008)	7.2	113	15%	6 months	Shelters/Temporary housing	Shibata^13^
Great East Japan (2011)	9	371	34.2%	5 months	Flooded shelters	Ueda^14^
		330	19.1%		Non-flooded shelters	
		1238	9.9%	2 years	Temporary housing	Shibata^15^
			12.7%	3 years		
			13.5%	4 years		
Kumamoto (2016)	6.5, 7.3	1673	10.6%	1-4 weeks	Shelters	Sato^16^
		42	14.3%	3 weeks	Shelters	Onishi^17^
		65	18.5%	8 months	Temporary housing	
		74	12.2%	19 months	Temporary housing	
Hokkaido East Iburi (2018)	6.7	195	9.7%	1-9 weeks	Shelters	Kamada^18^

*N/A,* Not applicable.

**Fig 1:**
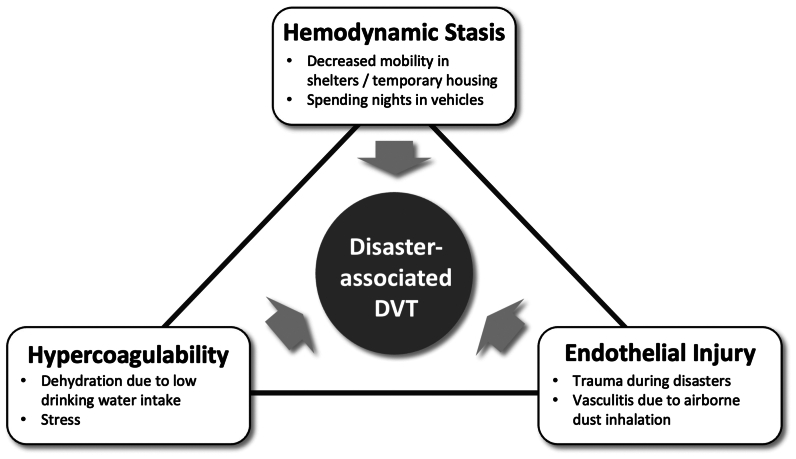
Virchow's triad and disaster-associated deep vein thrombosis (DVT). Hemodynamic stasis is associated with decreased mobility in evacuation shelters and temporary housing, as well as spending nights in vehicles. Hypercoagulability may arise secondary to dehydration owing to shortages of food and water, along with sympathetic nervous system activation under stress. Endothelial injury can result from trauma to the legs during disasters. Additionally, airborne dust containing silicon may trigger antineutrophil cytoplasmic antibody-associated vasculitis, potentially leading to endothelial damage.

General risk factors for DVT include advancing age, obesity, pregnancy, use of oral contraceptive medicine, diabetes, malignant tumor, congestive heart failure, chronic obstructive pulmonary disease, and a prior history of VTE.^20^ During disasters, DVT development is influenced by a combination of individual risk factors and adverse environmental conditions in evacuation shelters. The pathophysiology of the disaster-associated DVT can be understood through Virchow's triad: hemodynamic stasis, hypercoagulability, and endothelial injury (Fig 1). Mobility is often restricted in overcrowded shelters as compared with normal daily life.^14^ Risk further increases when evacuees sleep in vehicles, limiting mobility and leg stretching, contributing to hemodynamic stasis. Additionally, food and water supply shortages occur in evacuation shelters, and evacuees often hesitate to use temporary toilets owing to poor hygiene, leading to dehydration, an important factor in hypercoagulability.^14^ Stress-induced sympathetic nervous system activation also promotes a hypercoagulable state.^10^ A study during the 2011 Great East Japan earthquake identified reduced urination frequency and sleeping in vehicles as independent risk factors for DVT.^21^ Traumatic injuries to the legs sustained during earthquakes or evacuations can result in endothelial injury, further increasing the risk of DVT. Lower leg trauma has been specifically identified as a risk factor in disaster settings.^22^ In clinical practice, Wells' score is commonly used to assess DVT risk; however, disaster scenarios require considering the unique environmental conditions in each shelter. Many cases of disaster-associated DVT are considered preventable through proper interventions and improvements in the shelter environments.

## The recent history of disaster medicine in Japan

Disaster medicine in Japan has evolved through the lens of several major seismic events. This had led to the founding and mission of the DCC initiative.

### The 1995 Great Hanshin-Awaji earthquake

This 7.3 magnitude intraplate earthquake struck near Osaka and Kobe, causing more than 6000 deaths from collapsed houses and fires. Cardiovascular diseases, such as a three-fold increase in myocardial infarctions, doubled stroke rates, and Takotsubo cardiomyopathy, were observed among evacuees,^23^ suggesting the significant role of stress and poor nutrition. The use of triage tags to prioritize treatment and the establishment of the Disaster Medical Assistance Team (DMAT) followed.

### The 2004 Mid-Niigata Prefecture earthquake

This earthquake led to more than 60 deaths, with 11 cases of PE reported in Niigata Prefecture within 1 month. Most cases were associated with evacuees staying overnight in their vehicles,^5^ prompting widespread recognition of disaster-associated VTE in Japan. The DVT incidence was reported at 31.8% (Table II), reflecting the association with PE cases.

### The 2011 great East Japan earthquake

The Great East Japan earthquake triggered a massive tsunami, causing more than 15,000 deaths and a nuclear power plant accident. DVT incidence was 34.2% in tsunami-flooded areas, higher than in nonflooded areas (Table II). The shortage of food, water, and usable toilets contributed to dehydration, increasing VTE risk. Airborne dust from the tsunami-deposited sludge led to pneumonia and antineutrophil cytoplasmic antibody-associated vasculitis,^24^ further increasing DVT incidence.

### The 2016 Kumamoto earthquakes

Two consecutive earthquakes caused more than 200 fatalities. The Kumamoto Earthquakes Thrombosis and Embolism Protection Project conducted mobile venous screening in shelters,^16^ identifying approximately 10 PE cases, including 1 death from someone who slept in their vehicle. DVT incidence in shelters was 10.6% to 14.3% (Table II), slightly lower than in previous earthquakes. The DCC supported the Kumamoto Earthquakes Thrombosis and Embolism Protection Project's efforts.

### The 2018 Hokkaido East Iburi earthquake

This earthquake led to 42 fatalities. The DCC provided medical support to prevent VTE, including DVT screening, compression stockings distribution, education on VTE, and promoting cardboard beds. The incidence of DVT in shelters was 9.7% (Table II), with variations depending on the shelter environment, highlighting the importance of improving shelter conditions.^18^

### Establishment of the DCC in the Japanese Society of Phlebology

The DCC was established in 2016 as a committee affiliated with the Japanese Society of Phlebology. Its primary objectives are to (1) raise awareness and promoting the prevention of VTE, (2) provide ultrasound (US) screening for DVT among high-risk evacuees, (3) facilitate information sharing within disaster-affected areas, and (4) distribute compression stockings and provide instructions for proper use. The prevention of thrombus formation and progression is of paramount importance, including strategies for VTE prevention in evacuation shelters.

## The prevention of VTE during earthquakes

### The response to disasters in Japan

When disasters such as earthquakes occur, appropriate medical support is required at every phase of the response. During the acute phase, it is essential to ensure the availability of emergency medical care and the functioning of local health care systems. In the subacute to chronic phases, managing health conditions in evacuation shelters and restoring affected hospitals becomes critical.

Several organizations are involved in disaster medical support. First, DMAT is dispatched to the disaster area within 48 hours to initiate emergency medical activities, focusing on acute phase response, including on-site triage and emergency treatment, support for local hospitals, and transportation of patients to regional or wider-area medical facilities if necessary. After the acute phase, the Japan Medical Association Team, organized by the Japan Medical Association, takes over DMAT activities and provides mid- to long-term medical support, including public health services and coordination with local governments and medical associations. Additionally, other organizations involved in disaster medical response include the Self-Defense Forces, the local public health center, the Disaster Psychiatric Assistance Team, the Disaster Health Emergency Assistance Team, and the Japan Disaster Rehabilitation Assistance Team. The DCC plays a key role in preventing disaster-associated VTE through DVT screening, distributing compression stockings, promoting VTE awareness, and encouraging the use of cardboard beds to improve shelter environments.

### Education for evacuees about disaster-associated VTE

Educating evacuees in shelters about disaster-associated VTE is essential. Since the 2004 Niigata Prefecture earthquake, public awareness of VTE has increased; however, detailed knowledge, such as associated risks and prevention methods, remains limited. Even among physicians, familiarity with the specifics of disaster-associated VTE is not widespread. The DCC has placed a strong emphasis on educating evacuees by promoting preventive exercises and providing instructions on the use of compression stockings if needed. It also raises awareness about the risks of disaster-associated VTE, including the dangers of spending nights in vehicles. The DCC is engaged in disseminating educational resources on disaster medicine, targeted toward both the general public and health care professionals, through the Japanese Society of Phlebology website. Because disasters are not preventable and often large scale, preparation is crucial. Sharing accurate and practical information with both the general medical community and the public is essential for effective disaster response and VTE prevention.

### The algorithm for DVT screening

**Fig 2:**
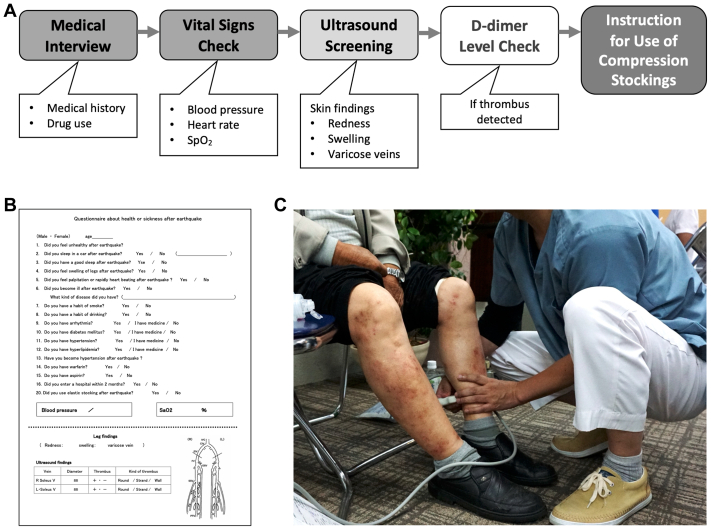
Deep vein thrombosis (DVT) screening in evacuation shelters. **(A)** During the medical interview, examinees complete a questionnaire to document their medical history and current medication use. Vital signs such as blood pressure, heart rate, and saturation of percutaneous oxygen are also recorded. Ultrasound (US) examination is used to screen the deep veins of the lower legs, and a visual inspection is conducted to assess for skin abnormalities. If a venous thrombus is identified, D-dimer levels are measured via blood testing. Compression stockings are then provided along with instructions for proper use. **(B)** Medical information obtained during the interview, along with US findings, is recorded on the screening sheet. If a thrombus is detected, its anatomical location, characteristics, and mobility are also documented. **(C)** Examinees remain seated during the US examination, which primarily focuses on screening the deep veins of the lower legs.

DVT screening is conducted for evacuees in shelters, particularly targeting high-risk individuals such as the elderly, those with low mobility, and those with a medical history of cancer, to maximize screening efficacy. An example of a screening algorithm flowchart is shown in Fig 2, *A*.^16^^,^^18^ During the medical interview, vital signs—including blood pressure, heart rate, and saturation of percutaneous oxygen—are recorded, and drug use as well as medical history are reviewed. US examination of the deep veins in the lower legs is then performed, along with visual inspection for skin findings such as redness, swelling, and varicose veins. If a venous thrombus is detected, the D-dimer level is measured via blood test. If the D-dimer level exceeds 2.0 μg/mL^16^ or if extensive DVT is present, the patient is referred to a specialized hospital for further evaluation and treatment. All high-risk evacuees who underwent DVT screening are provided with compression stockings and instructed on their proper use, unless contraindicated. Medical information on evacuees is recorded on screening sheets (Fig 2, *B*).

### The efficacy of US examination for DVT detection

Simple and prompt diagnosis is essential for DVT screening in evacuation shelters. US examination is minimally invasive and well-suited for this purpose. Battery-powered portable US devices are especially advantageous, because access to electricity may be limited in disaster settings. US screening primarily targets the deep veins in the lower legs (Fig 2, *C*). Findings such as anatomical location, thrombus characteristics, and its mobility are also recorded on screening sheets (Fig 2, *B*). Owing to space constraints in shelters, providing private rooms for examination is often not feasible, making it impractical to extend scanning to the whole leg. However, a previous study has shown that more than 90% of massive PEs originate in the deep veins of the lower legs based on autopsy findings.^25^ Additionally, approximately 70% of DVTs among positive cases were found in the soleal veins,^12^ and dilation of the soleal vein has been reported as a risk factor for DVT.^26^ Consequently, owing to the lack of private spaces and the evidence supporting lower leg screening, US examinations are restricted to the calf to popliteal region at the beginning of examination.

### Compression stockings for DVT prevention

Compression stockings increase venous blood flow velocity and reduce venous congestion in the lower extremities by decreasing the total cross-sectional area of the veins. This is a simpler, more practical, and cost-effective method compared with other preventive interventions. In clinical practice in Japan, perioperative PE has decreased by nearly one-half since the widespread adoption of early mobilization and compression stocking use.^27^ Conversely, clinical guidelines from the American College of Physicians do not recommend compression stockings for VTE prevention in hospitalized patients, because their end points have shifted to fatal or symptomatic DVT.^28^ Owing to these different perspectives, the effectiveness of compression stockings for VTE prevention in evacuation shelters may be controversial. However, the thrombogenicity of Japanese and other East Asians is inherently much lower than that of Caucasians or Africans, largely owing to differences in coagulation factor profiles,^29^ which can partly explain the divergence in the Japanese and the United States guidelines. Given the demonstrated efficacy of compression stockings in reducing VTE risk in airplane passengers^30^ and their success in decreasing VTE incidence among patients in Japan, it is reasonable to follow the Japanese guidelines and to expect that compression stockings may provide some benefit in shelter environments, especially considering their cost effectiveness.

The DCC collaborates with medical institutions and local governments throughout Japan to establish stockpiles of compression stockings for future disasters. These stockings are transported to affected areas and distributed alongside DVT screening, with proper instructions for use. Coordination with local medical institutions, DMAT, and Japan Medical Association Team staff is necessary for efficient transport and distribution of compression stockings. In addition, the DCC engages in public awareness efforts, including the creation of educational pamphlets on VTE prevention and lower limb exercises, in cooperation with public health centers and local authorities.

### The effectiveness of cardboard beds for improving the shelter environment

**Fig 3:**
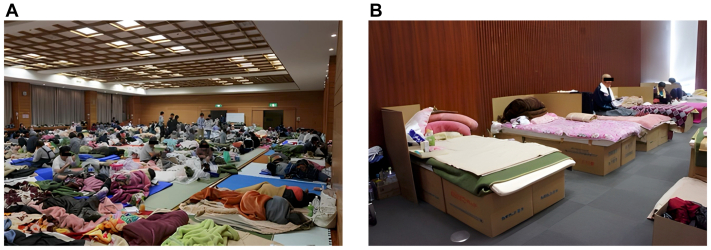
Sleeping environment in evacuation shelters in Japan. **(A)** Huddle sleeping style. Mattresses are placed directly on the floor, requiring evacuees to sleep in close proximity to the ground. This setup is unhygienic and demands considerable muscular effort to stand up, particularly for elderly individuals, resulting in decreased mobility. **(B)** Cardboard beds are constructed from multiple pieces of cardboard that can be easily assembled without special equipment. These beds offer a more hygienic sleeping arrangement with better thermal insulation by elevating evacuees off the floor.

During the 2011 Great East Japan earthquake, it was noted that poor shelter environments contribute to an increased risk of DVT.^14^ Traditionally in Japan, evacuees slept on mattresses placed directly on the floor, known as huddle sleeping style (Fig 3, *A*). However, this practice had several problems. Sleeping close to the floor is unhygienic and requires significant muscular effort to stand up, especially for elderly, reducing mobility and the heightening risk for DVT. Historically, underground evacuation during the air attacks on London in 1940 caused many deaths owing to PE. Since then, the use of cots with some height that are raised from the floor during disasters has become common in Western countries as a preventive measure.

In recent years, cardboard beds have gained attention in Japan. The Cabinet Office guidelines for evacuation shelter management recommend early introduction of cardboard beds, as well as compression stockings. Cardboard beds are assembled by combining multiple pieces of cardboard to create a cot-like structure. They are easy to set up, hygienic, and offer good thermal insulation by elevating sleepers off the floor (Fig 3, *B*). This elevation facilitates standing and improves mobility, especially in the elderly. Additionally, cardboard beds are more affordable and easier to transport than conventional cots. Previous studies have shown that the incidence of disaster-associated DVT begins to increase approximately 1 week after an earthquake.^16^^,^^18^ Therefore, the prompt introduction of cardboard beds is strongly recommended to improve mobility, enhance hygiene, and reduce the risk of DVT.

### The management of DVT in the chronic phase

For evacuees whose homes were destroyed, temporary housing is typically constructed during the acute phase of a disaster. Depending on the scale of the disaster, evacuees may move into temporary housing within 1 to 3 months after the event. Although it is expected that the incidence rate of DVT would decrease owing to improved living conditions compared with evacuation shelters, more than 10% of evacuees were found to have a DVT, even in temporary housing after the 2011 Great East Japan earthquake and the 2018 Kumamoto earthquake (Table II).

The persistence of DVT in temporary housing is largely attributed to an inactive lifestyle. After the 2011 Great East Japan earthquake, decreased social interaction among residents was reported, and opportunities to go outside were fewer than usual, leading to decreased mobility and a higher risk of DVT. In the chronic phase, mental stress also becomes a significant concern, because it can worsen lifestyle habits such as smoking and alcohol consumption, further reducing physical activities. To address these issues, it is crucial to provide exercise guidance and conduct follow-up DVT screening in temporary housing. These efforts are expected to be managed and coordinated by the DCC.

## Conclusions

Japan has experienced numerous major earthquakes over the last century, and substantial knowledge about disaster-associated VTE has been accumulated, particularly in the last two decades. It has become clear that not only individual risk factors, but also the evacuation environment play a significant role in the development of VTE. Therefore, it is essential to establish systems that eliminate these aggravating conditions. In the future, Japan is expected to face the Nankai Trough earthquake, a devastating event that could occur off the southern coast of the country. To prevent VTE and other health complications during such disasters, collaboration with local governments and medical institutions will be essential. This preparation includes stockpiling compression stockings and cardboard beds and ensuring efficient systems for their rapid distribution during emergencies. Moreover, sharing appropriate and practical knowledge with health care professionals is crucial for implementing effective VTE countermeasures. Continued education and preparedness will play a key role in mitigating the health risks associated with future disasters.

## Author Contributions

Conception and design: KK, EF

Analysis and interpretation: KK

Data collection: KK, EI, DU, MM, KH, SU, NA

Writing the article: KK, EF

Critical revision of the article: KK, EF, EI, DU, MM, KH, SU, NA

Final approval of the article: KK, EF, EI, DU, MM, KH, SU, NA

Statistical analysis: Not applicable

Obtained funding: Not applicable

Overall responsibility: KK

## Funding

The Department of Advanced Treatment and Prevention for Vascular Disease and Embolism, Niigata University Graduate School of Medical and Dental Sciences, to which Dr. Kazuhiko Hanzawa belongs, receives financial support from Kijokai Social Medical Corporation, ONYONE, Co., Ltd, Thanko, Inc., NANO SUI, Co., Ltd., Shisei datum, JMR Co., Ltd, Aomi Precision Co., Ltd.

## Disclosures

None.
